# Impact of the Trump Administration's Expanded Global Gag Rule Policy on Family Planning Service Provision in Ethiopia

**DOI:** 10.1111/sifp.12196

**Published:** 2022-05-31

**Authors:** Elizabeth A. Sully, Solomon Shiferaw, Assefa Seme, Suzanne O. Bell, Margaret Giorgio

**Affiliations:** ^1^ Guttmacher Institute New York NY 10038 USA; ^2^ School of Public Health Addis Ababa University Addis Ababa Ethiopia; ^3^ Department of Population Family and Reproductive Health Johns Hopkins Bloomberg School of Public Health Baltimore MD 21205 USA

**Keywords:** GGR, Health policy

## Abstract

The Global Gag Rule (GGR) makes non‐U.S. nongovernmental organizations (NGOs) ineligible for U.S. Government global health funding if they provide, refer, or promote access to abortion. This study quantitatively examines the impacts of the GGR on family planning service provision in Ethiopia. Using a panel of health facilities (2017–2020), we conduct a pre–post analysis to investigate the overall changes in family planning service provision before and after the policy came into effect in Ethiopia. Our pre–post analyses revealed post‐GGR reductions in the proportions of facilities reporting family planning provision through community health volunteers (−5.6, 95% CI [−10.2, −1.0]), mobile outreach visits (−13.1, 95% CI [−17.8, −8.4]), and family planning and postabortion care service integration (−4.8, 95% CI: [−9.1, −0.5]), as well as a 6.1 percentage points increase in contraceptive stock‐outs over the past three months (95% CI [−0.6, 12.8]). We further investigate the impacts of the GGR on facilities exposed to noncompliant organizations that did not sign the policy and lost U.S. funding. We do not find any significant additional impacts on facilities in regions more exposed to noncompliant organizations. Overall, while the GGR was slow to fully impact NGOs in Ethiopia, it ultimately resulted in negative impacts on family planning service provision.

## INTRODUCTION

**FIGURE 1:**
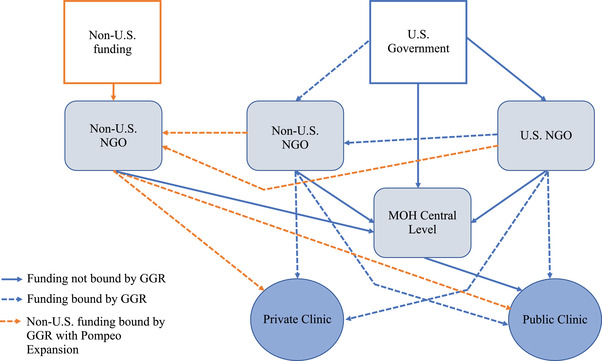
U.S. Government global health assistance bound by the Global Gag Rule and later Pompeo Expansion NOTE: Squares represent donors, curved rectangles are funding recipients, and circles represent the health system. With the Pompeo Expansion, U.S. and non‐U.S. NGOs that complied with the policy had their non‐U.S. funding then subject to the terms of the GGR. As a result, non‐U.S. NGOs (far left) had all their funding streams impacted by the policy as well due to their compliance. Prior to this expansion, all orange arrows were not bound by the conditions of the GGR. SOURCE: Adapted from Schaaf et al. (2019) to account for the Pompeo Expansion.

The Global Gag Rule (GGR) is a United States (U.S.) Government policy that deems non‐U.S. nongovernmental organizations (NGOs) ineligible for U.S. Government global health funding if they use their own funds to provide, refer for, or promote access to abortion. Since first implemented by the Reagan administration in 1984, the GGR has been rescinded by all Democratic presidents and reinstated by all Republican presidents (Lo and Barry 2017). In January 2017, the Trump administration reinstated an expanded GGR under the new name “Protecting Life in Global Health Assistance” (The White House 2017). While prior iterations of the GGR only applied to family planning assistance (∼$600 million USD annually), the Trump administration's GGR applies to all global health funding (∼$12 billion USD annually) (U. S. Government Accountability Office 2020). In May 2019, the GGR was further expanded through new guidance issued by the Department of State (Pompeo 2019). Prior to this so‐called “Pompeo Expansion,” the GGR only impacted organizations that received U.S. Government funding. With the expansion, all subgrantees of compliant organizations became subject to the policy, including those who were subgranted non‐US Government funds (Figure 1). All affected non‐U.S. NGOs were required to comply with the GGR, regardless of national abortion laws, until it was rescinded by the Biden Administration in January 2021 (The White House 2021).

When the GGR is operating, there are two possible outcomes for affected non‐U.S. NGOs. First, NGOs comply with the policy, requiring changes to current services, activities, and/or partnerships that were in violation of the GGR (e.g. providing abortions, information on or referrals for abortion, or advocating for abortion access). It has also been hypothesized that compliant organizations can overinterpret the policy, resulting in ceasing postabortion care services or not referring women to noncompliant organizations for services other than abortion (Schaaf et al. 2019). The second possible outcome is that NGOs refuse to comply with the policy and lose U.S. Government funding, as well as other funding sources and/or partnerships. This loss of funding can lead to decreases in staff, the types of services offered, clinic outreach efforts, and opening hours, as well as increases in wait times (Schaaf et al. 2019). Further, it is not only the services provided directly by noncompliant organizations that are impacted; while the Ministry of Health is exempt from the GGR, public health systems receive direct funding and technical support provided by non‐U.S. NGOs (Figure 1). As such, funding cuts for noncompliant organizations can impact service availability in both the public and private sectors. With both possible outcomes, it is hypothesized that changes in services from compliant and/or noncompliant organizations, in addition to a larger climate that impacts partnerships, advocacy, and public education, may lead to downstream impacts on women's reproductive health outcomes, including a decrease in contraceptive use and an increase in unplanned births and abortions (Giorgio et al. 2020).

Previous cross‐national research has supported these hypothesized causal pathways, finding that prior iterations of the GGR resulted in decreases in contraceptive use and increases in abortions and pregnancies in countries receiving a greater share of U.S. family planning assistance (Bendavid, Avila, and Miller 2011; Brooks, Bendavid, and Miller 2019). Another study with a similar design found regional differences, with the likelihood of abortion increasing in more exposed countries in sub‐Saharan Africa and Latin America, and the Caribbean, but decreasing in South and Southeast Asia (van der Meulen Rodgers 2018). Further, a country‐specific study in Ghana found that contraception access declined and unintended pregnancies increased in rural areas when the GGR was in effect, resulting in an increase in abortions and unplanned births (Jones 2015).

Existing research on the Trump Administration's GGR is largely qualitative, drawing from interviews among NGO staff, service providers, and other key stakeholders and has not examined larger health system or population‐level impacts (PAI 2018, 2019; Ravaoarisoa et al. 2020; Tamang et al. 2020; Ushie et al. 2020). Qualitative reports and studies have documented changes in contraceptive service provision (including clinic closures and termination of mobile outreach services), losses of service integration, a weakened advocacy environment, and fractured partnerships and referral networks (Ravaoarisoa et al. 2020; Tamang et al. 2020; Ushie et al. 2020). One quantitative, quasi‐experimental study in Uganda found that in the early years of the policy implementation, there was a decrease in community health workers engaged in family planning activities (Giorgio et al. 2020). However, little is known about the multiyear impacts of the expanded GGR, the impact of the Pompeo expansion, or impacts in a context where abortion is broadly legal.

Ethiopia is an important context in which to study the impact of the GGR. Ethiopia has the fifth highest amount of U.S. Government funding subject to the GGR (U. S. Government Accountability Office 2020). In the fiscal year 2017, Ethiopia received $293 million (USD) from the U.S. Government for global health assistance, of which $32 million was for family planning and reproductive health (ForeignAssistance.gov 2020). U.S. Government global health assistance accounted for 35% of all development assistance for health received by Ethiopia in 2017 (Institute for Health Metrics and Evaluation 2021), and donor funding (including from the U.S. Government) accounted for 70% of Ethiopia's family planning budget (World Health Organization 2021). Given that U.S. Government funding is an essential component of Ethiopia's health system, GGR restrictions have the potential to drastically affect family planning service provision. Further, while only 10 prime grantee NGOs receive the majority of U.S. Government funding for family planning, HIV, and maternal/child health in Ethiopia, each NGO has as many as 20 subgrantees (PAI 2018). This makes Ethiopia additionally vulnerable to the impacts of the Pompeo Expansion.

GGR‐related U.S. Government funding constraints may counteract the Ethiopian government's efforts to expand women's access to family planning and safe abortion care (SAC). Ethiopia revised and expanded its abortion law in 2005 and has made considerable efforts to improve the availability of SAC (Bridgman‐Packer and Kidanemariam 2018; Ethiopia Ministry of Health 2006; Moore et al. 2016). Efforts to increase access to family planning services have resulted in modern contraceptive use more than doubling from 10% in 2005 to 25% in 2016 (Central Statistical Agency [Ethiopia] and ORC Macro 2006; CSA [Ethiopia] and ICF 2016). One key factor driving these improvements is NGOs’ role in expanding the provision of public sector services, including mobile outreach programs that provide long‐acting reversible contraception (LARCs), training of public health workers, and technical assistance to programs such as Health Extension Workers (HEWs) who provide essential services to under‐served rural populations (Olson and Piller 2013; PAI 2018). The critical role of NGOs and the Ethiopian government's concerted efforts to expand healthcare access illustrates the vulnerability of both public and private family planning services under the GGR.

The aim of this study is to assess the impact of the expanded GGR on family planning service delivery in Ethiopia using a panel of health facilities from 2017 to 2020. We conduct a pre–post analysis to assess changes in family planning services, including contraceptive provision, family planning outreach services, and integration with other sexual and reproductive health (SRH) services before and after the GGR came into effect. We further measure district‐level exposure to the GGR and use a quasi‐experimental difference‐in‐difference (DID) design to estimate the additional impacts of the policy in areas where NGOs not complying with the policy had been supporting SRH service provision.

## METHODS

### Data Sources and Sample

**FIGURE 2:**
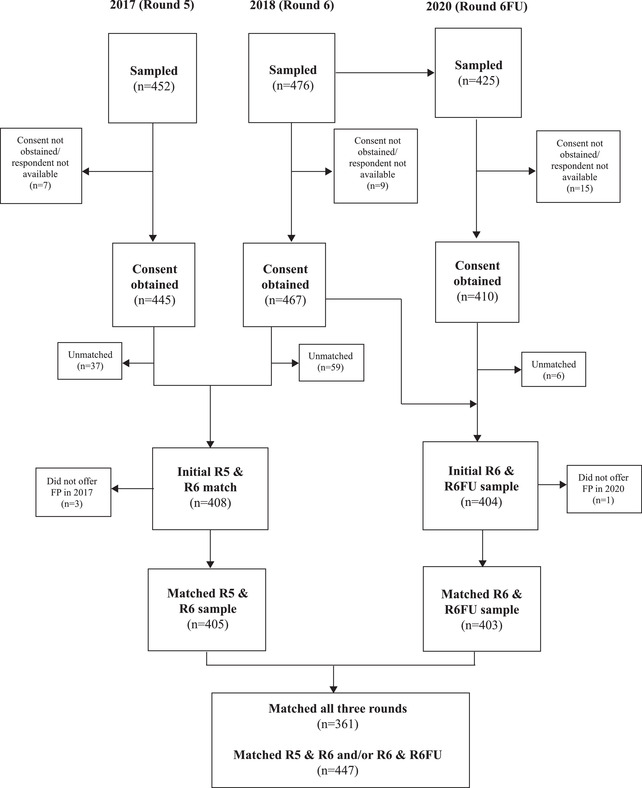
Flowchart of facility inclusion and exclusion

Data for this analysis come from the 2017/2018 Ethiopia Performance Monitoring and Accountability 2020 (PMA2020) surveys and a follow‐up 2020 GGR Panel survey. PMA2020 consists of annual surveys of women and family planning service delivery points (SDPs), whose sampling/survey methodology is described in detail elsewhere (Zimmerman et al. 2017). In brief, 221 enumeration areas (EAs) were selected using probability proportional to size sampling from urban/rural and region strata, and a nationally representative sample of women was selected from the EAs. The SDP sample is designed to represent health facilities that serve the female sample, including all public facilities that serve selected EAs, regardless of location, and a random sample of ≤3 private facilities within each EA. The 2020 GGR Panel survey attempted to reinterview all 2018 PMA facilities located in six study regions (Addis Ababa, Afar, Amhara, Oromia, SNNPR, and Tigray). The PMA2020 sample included 452 facilities in 2017 and 476 in 2018, and 425 facilities were eligible for the GGR Panel survey in 2020 (Figure 2). We excluded facilities that did not provide family planning or were not surveyed in at least two rounds, resulting in a final sample of 447 (361 included in all three rounds). The GGR 2020 panel survey was completed in March, immediately prior to COVID‐related disruptions in Ethiopia.

To measure exposure to the GGR, we conducted in‐person meetings with 15 key stakeholders in March 2018 and email follow‐up conversations between August and October 2020. Key stakeholders included prime and subgrantee non‐U.S. NGOs, donor organizations and governments, and the Ministry of Health. Stakeholders provided information on GGR compliance, GGR‐related changes in services and/or funding, any stopgap funding provided/received from donor governments, and, when non‐U.S. NGOs were providing SRH services, the scope, and geographic coverage of their SRH service delivery before and after GGR reimplementation. The two largest noncompliant NGOs provided detailed monitoring and evaluation data on the districts and number of facilities that were served by their U.S.‐funded programs prior to the GGR, as well as after their U.S. Government funding ended.

This study received ethical approval from the Institutional Review Boards of the Guttmacher Institute, Johns Hopkins Bloomberg School of Public Health and Addis Ababa University.

### Measures: Exposure

Using the information provided by stakeholders, we mapped out GGR compliant and noncompliant organizations in 2018 and 2020 (Table 1). While the GGR came into effect in May 2017(U. S. Government Accountability Office 2020), the policy rollout in Ethiopia was gradual due to staggered end dates of existing contracts and funding agreements. Key stakeholders revealed that the two largest noncompliant NGOs either were not asked to comply with the GGR until 2019 or received complete stopgap funding for 2018. However, this stopgap funding was unable to fully compensate for lost U.S. Government funding in subsequent years, and by 2020 both large noncompliant organizations had lost funding. The result of these changes was reductions in services: a youth program reduced geographic coverage, a mobile outreach program scaled back, a technical support program to the public sector ended, and overhead funding was lost.

**TABLE 1:** Compliance or noncompliance with the Global Gag Rule policy across non‐U.S. NGOs in Ethiopia, 2018 and 2020

	2017	2018	2020
Noncompliant organizations			
	NA	One large NGO did not sign and lost U.S. funding; received 100% stopgap funding from other donors for all of 2018 One large NGO retained U.S. funding through the end of 2018	One large NGO did not retain full stopgap funding; scaled back a youth program on strengthening health systems from 24 to 6 woredas One large NGO lost U.S. funding, and only received partial stopgap funding from other donors: (1) scaled back outreach program focused on LARC and permanent methods in rural areas, serving fewer woredas or fewer facilities within a woreda, (2) ended the program on providing technical support to the public sector, and (3) lost overhead support
Compliant organizations			
	NA	One large regional development association signed; impacted the Health Extension Workers program One small NGO signed; ended the safe abortion referral program for students at one university	All regional development associations signed; impacted Health Extension Workers program Pompeo Expansion resulted in widespread compliance by 2020

NOTE: NA, not applicable.

The number of organizations complying with the policy changed greatly between 2018 and 2020 (Table 1). In 2018, while several foreign NGOs signed the policy and complied with its terms, we only identified two organizations whose compliance required a change in previously provided services. The first was a small subgrantee organization, which was required to suspend referrals for safe abortion services provided at one university. The second was a regional development association; development associations are parastatal NGOs, mandated to implement development activities within the region. Critically, they are responsible for the training of HEWs, who are government public health employees that link communities to the health system, including providing referrals for SAC. While there are many regional developmental associations in Ethiopia, the program and contract timing meant that only one was required to sign in 2018. By 2020, more regional development associations had been asked to comply with the policy, and the Pompeo Expansion resulted in widespread compliance across all regions. This removed all geographic variation in exposure, and by 2020 all facilities were classified as exposed to the GGR. In order to make causal claims about the impact of the GGR on family planning service delivery in Ethiopia, a comparison group or counterfactual is needed. In the absence of an appropriate counterfactual, we look broadly at the changes over time, comparing service delivery levels prior to the GGR (2017) to those after GGR came into effect (2018–2020).

We initially hypothesized that noncompliant organizations might have a larger impact on service delivery outcomes compared to compliant organizations due to loss of funding. As such, we also constructed a separate exposure variable that focuses only on organizations that refused to sign and comply with the policy (hereafter referred to as noncompliance exposure). Facilities were classified as exposed to noncompliant organizations if they resided in a woreda (i.e., district) where services were reduced after U.S. Government funding was lost and no stopgap funding was secured. Overall, 22% of facilities were classified as exposed to the organization not complying with the GGR. Given no GGR‐related changes to services due to noncompliance had occurred by 2018, the preperiod is classified as 2017‐2018, and the postperiod is 2020.

### Measures: Outcomes

Our main outcome variables reflect three areas of family planning services: contraceptive provision, family planning outreach services, and family planning service integration. For contraceptive provision, we hypothesized that fewer facilities would be able to provide LARCs given NGOs’ role in LARC provision, both directly and through mobile outreach and technical assistance. As such, we measured whether facilities offered IUDs or implants. We also measured short‐term contraceptive commodity stock‐outs. Stock‐outs were determined based on which modern methods a facility reported they normally offered (IUDs, implants, injectables, pills, male/female condoms, emergency contraception) and whether a stock‐out of one or more methods was reported in the past three months.

We hypothesized that the GGR would result in declines across several areas of family planning outreach services, as NGO's provide technical assistance, mobile outreach, and support for Community Health Volunteers (CHVs). We measured whether family planning was provided through CHVs and the number of CHVs supported. We also measured whether any mobile outreach visits had occurred at a facility in the past 12 months, the number of mobile outreach visits, the number of clients served and whether LARCs were provided through the mobile outreach. The latter two variables were only measured for 2018 and 2020. We also constructed two dichotomous variables for whether a facility received NGO support generally and specifically for CHVs. (Information on NGO support was only measured in 2018 and 2020.)

Due to the expanded scope of the GGR, we hypothesized that the policy would lead to lower levels of facility‐reported integration of family planning services with other SRH services. Therefore, we included two indicators for whether a facility reported providing integrated family planning with HIV and/or postabortion care (PAC) services.

We also measured potential confounding factors, including facility type (hospital, health center, health post, health clinic, and pharmacy/retail), and district‐level modern contraceptive prevalence rate (mCPR), measured using the corresponding PMA female surveys in each year.

### Statistical Analysis

We conducted two complementary analyses to estimate GGR‐related changes in family planning service delivery outcomes. Our initial study design intended to compare regions of the country that were more and less exposed to the GGR in order to estimate the impacts of the policy. However, following the Pompeo Expansion, no regional variation remained, and there was no suitable control group. As such, our main set of analyses is limited to a pre–post design, comparing 2017 (pre‐GGR) to 2018–2020 (post‐GGR). In addition, we also investigate the impacts of noncompliance exposure using a quasi‐experimental DID design, given that geographical variation in this exposure remained throughout the study period. The DID does not have these same causal limitations and, moreover, can illustrate the additional impacts specific to noncompliant organizations' loss of funding due to the GGR.

We first assessed the overall impacts of the policy before and after the GGR came into effect using a pre–post analysis. Multivariable regression models were fitted for each outcome using the following equation (1):

(1) $$Y_{ij}=\beta_{0+}\beta_{1}S_{j}+\beta_{2}F_{i}+\beta_{3}C_{ij}+\beta_{4}R_{i}+\varepsilon_{ij}.$$

The outcome of interest (*Y_ij_*) is estimated for facility *i* during survey round *j*. The model includes year (*S*), facility type (*F*), district mCPR (*C*), and region fixed‐effects (*R*). Robust standard errors accounting for clustering at the EA level were calculated. We calculated postestimation predicted probabilities for the differences between 2017 and 2020. Dichotomous outcomes were fitted using logit models, continuous outcomes using ordinary least squares regression.

As causal inference is limited using the pre–post analysis, we investigated whether the pre–post results were a continuation or deviation from pre‐GGR trends. We constructed indicators for all study outcomes using cross‐sectional PMA data from 2014 to 2016, and we plotted trends in these outcomes against the results from our panel analysis. Our pre–post estimates from 2017 to 2020 are not perfectly comparable to 2014–2016 PMA data given sample differences (nationally representative cross‐sections vs panel) and that pre–post estimates are predicted probabilities generated from models that control for facility type, mCPR, and region.

The impact of noncompliance exposure was estimated using a DID approach. First, we assessed whether there were differences in facility characteristics, outcome indicators, and community‐level characteristics by noncompliance exposure status at baseline. Facility‐ or community‐level characteristics that varied significantly by exposure status were adjusted for in our multivariable analysis. Multivariable regression models were fitted for each outcome using equation (2). The DID model includes the exposure variable (*E*), pre‐/post‐GGR indicator (2017/2018 vs. 2020) (*T*), and an interaction of these variables (E*T). The key measure of the GGR impact of noncompliance exposure is the estimated DID from the interaction term in the model. Region fixed‐effects (*R*) are excluded from the model due to collinearity with noncompliance exposure.

(2) $$Y_{ij}=\beta_{0}+\beta_{1}E_{i}+\beta_{2}T_{j}+\beta_{3}\left(E_{i}*T_{j}\right)+\beta_{4}F_{i}+\beta_{5}C_{ij}+\beta_{6}S_{j}+\varepsilon_{ij}.$$

The most important assumption of the DID model is that there were no differences in the trends between exposure groups prior to the treatment (GGR policy) coming into effect. We assess for parallel trends by GGR exposure using cross‐sectional PMA data from 2014 to 2018 and the GGR panel for 2020. Parallel trend analyses are conducted on the key outcomes that were included in prior PMA survey rounds (provides IUDs, provides implants, stock‐outs of any method in the past three months, provides family planning through CHVs, number of CHVs, any mobile outreach in the past 12 months, number of mobile outreach visit, providing integrated family planning with HIV, and integrated family planning with PAC services).

All analyses were performed using Stata version 15.0 (StataCorp. 2017).

## RESULTS

**FIGURE 3:**
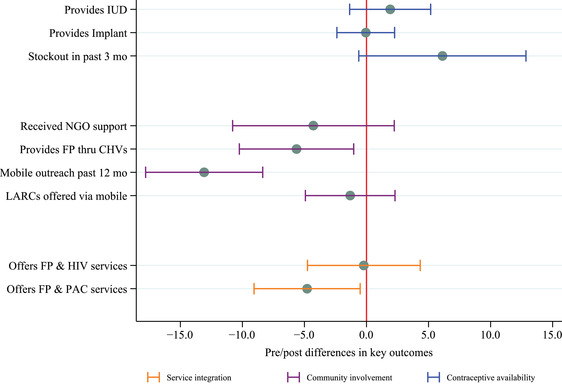
Pre–Post estimates of the impact of the GGR on family planning service delivery in Ethiopia, 2017–2020

**FIGURE 4:**
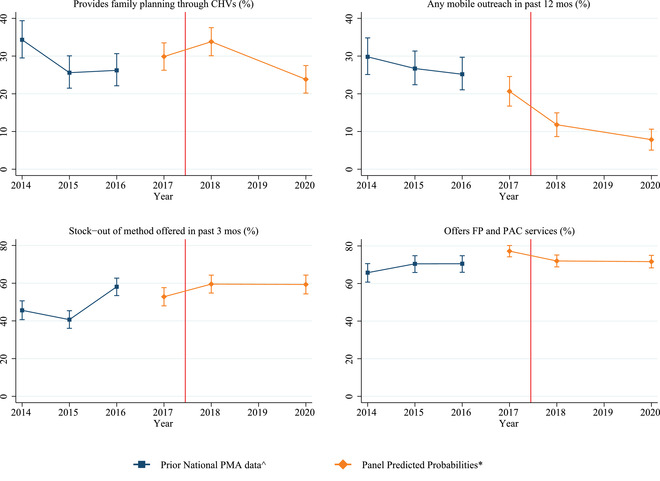
Pre‐GGR trends in family planning service outcomes that significantly changed following the reinstatement of the GGR, 2014–2020

In assessing the overall changes during the time when the GGR was in effect, we find reductions from pre‐ to post‐GGR in the proportion of facilities that provided family planning through CHVs (−5.6, 95% CI [−10.2, −1.0]), the proportion with any mobile outreach visits in the past 12 months (−13.1, 95% CI [−17.8, −8.4]), and the proportion reporting integration of family planning and PAC services (−4.8, 95% CI [−9.1, −0.5]) (Figure 3, Supplemental Table S1). We also observed a marginally significant increase in stock‐outs of any method in the past three months of 6.1 percentage points (95% CI [−0.6, 12.8], *p* < 0.075). There were no clear trends prior to the introduction of the GGR for the provision of family planning through CHVs, stock‐outs, and integration of family planning and PAC (Figure 4). The proportion of facilities receiving any mobile outreach in the past 12 months has been declining since 2014. While the decline between 2014 and 2017 is not statistically different, the pre–post GGR trends do represent a statistically significant decline.

To investigate the additional impacts of facilities being exposed to organizations that did not comply with the policy, we first assess the balance between exposure arms at baseline among the facilities interviewed in 2017 that we have follow‐up data for (*N* = 405) (Table 2). There were no differences in facility type or managing authority by noncompliance exposure at baseline. Due to the geographic nature of noncompliance exposure, we observed differences by region. There were also statistically significant differences in exposure status for mCPR (30% vs. 25%, *p* < 0.001), and a larger proportion of exposed facilities provided family planning through CHVs (39% vs. 27%, *p* < 0.021). Our DID estimator for noncompliance exposure (2017‐2018 vs. 2020) was not statistically significant for declines in contraceptive stock‐outs, LARC availability, NGO support, support for CHVs, any mobile outreach, or service integration (Table 3). We did find that, among facilities that received at least one mobile outreach visit, exposed facilities experienced an increase post‐GGR in the number of mobile outreach visits compared to unexposed facilities (3.69, 95% CI [0.4,7.0]).

**TABLE 2:** Baseline differences between service delivery points by exposure to organizations noncompliant with the GGR in Ethiopia, 2017

	Total		Exposed		Unexposed		
	(*N* = 405)		(*N* = 91)		(*N* = 314)		*p*‐value
*Facility characteristics*							
Facility type, *n* (%)							0.314
Hospital	97	(24%)	19	(21%)	78	(25%)	
Health center	192	(47%)	40	(44%)	152	(48%)	
Health post	85	(21%)	26	(29%)	59	(19%)	
Health clinic	16	(4%)	4	(4%)	12	(4%)	
Pharmacy/retail	15	(4%)	2	(2%)	13	(4%)	
Region, *n* (%)							<0.001
Addis Ababa	27	(7%)	0	(0%)	27	(9%)	
Afar	20	(5%)	7	(8%)	13	(4%)	
Amhara	87	(21%)	28	(31%)	59	(19%)	
Benishangul‐Gumuz	11	(3%)	0	(0%)	11	(4%)	
Dire Dawa	2	(0%)	0	(0%)	2	(1%)	
Gambella	5	(1%)	0	(0%)	5	(2%)	
Harari	3	(1%)	0	(0%)	3	(1%)	
Oromia	96	(24%)	15	(16%)	81	(26%)	
Somali	7	(2%)	0	(0%)	7	(2%)	
SNNP	90	(22%)	23	(25%)	67	(21%)	
Tigray	57	(14%)	18	(20%)	39	(12%)	
Managing authority, *n* (%)							0.605
Government	372	(92%)	85	(93%)	287	(91%)	
NGO	3	(1%)	0	(0%)	3	(1%)	
Private	30	(7%)	6	(7%)	24	(8%)	
*Community‐level characteristics*							
Modern contraceptive prevalence rate (mCPR) by district, mean (range)	0.26	(0.00–0.67)	0.30	(0.03–0.59)	0.25	(0.00–0.67)	<0.001
Proportion of facilities offering IUD by EA, mean (range)	0.62	(0.00–1.00)	0.60	(0.00–1.00)	0.63	(0.00–1.00)	0.485
*Contraceptive availability*							
Modern methods offered, *n* (%)							
Sterilization (male or female)^a^	88	(23%)	18	(20%)	70	(23%)	0.548
IUDs^a^	263	(67%)	57	(64%)	206	(68%)	0.437
Injectables	391	(97%)	90	(99%)	301	(96%)	0.162
Implants^a^	356	(91%)	78	(88%)	278	(92%)	0.166
Pills	400	(99%)	90	(99%)	310	(99%)	0.894
Condoms (male or female)	395	(98%)	89	(98%)	306	(97%)	0.850
Stock‐out of any method offered last 3 months,^b^ *n* (%)	215	(53%)	49	(54%)	166	(53%)	0.869
*Family planning outreach services*							
Provides family planning through CHVs,^a^ *n* (%)	115	(29%)	35	(39%)	80	(27%)	0.021
Number of CHVs supported to provide FP,^e^ mean(range)	70	(1–630)	65	(1–630)	73	(1–310)	0.447
Any mobile outreach visit in the past 12 months,^a^ *n* (%)	79	(21%)	24	(27%)	55	(19%)	0.085
Number of mobile outreach visits in the past 12 months,^e,a^ mean(range)	2.5	(1–24)	2	(1–5)	3	(1–24)	0.572
Service integration							
Offers FP and HIV services, *n* (%)	360	(89%)	79	(87%)	281	(89%)	0.474
Offers FP and PAC services,^d^ *n* (%)	300	(77%)	65	(73%)	235	(78%)	0.322

Abbreviations: CHV, Community Health Volunteers; EA, enumeration area; FP, family planning; GGR, Global Gag Rule; IUD, intrauterine device; NGO, nongovernmental organization; PAC, post‐abortion care.

^a^ Excludes pharmacies and retail outlets.

^b^ Any stock‐out of any family planning method in the past three months. The summary measure of stock‐outs (IUDs, injectables, implants, pills, male condoms, female condoms, emergency contraception).

^c^ Only among SDPs that reported providing FP through HEWs and provided a valid, numeric response (*N* = 107, 35 exposed and 72 unexposed).

^d^ Excludes health clinics, pharmacies, and retail outlets.

^e^ Only among SDPs that reported receiving any mobile outreach and provided a valid, numeric response (*N* = 79, 24 exposed and 55 unexposed).

**TABLE 3:** Difference‐in‐Differences estimates of the impact of exposure to organizations’ noncompliant with the GGR on facilities in Ethiopia, 2017–2020

	Estimated adjusted proportion/mean in preperiod(2017–2018)		Estimated adjusted proportion/mean in postperiod(2020)		Impact of the GGR Policy		
	Exposed	Unexposed	Exposed	Unexposed	Difference in difference	95% CI	*p*‐value
Contraceptive availability							
Offers intrauterine devices^e^	70.37	69.24	65.94	68.23	−3.42	[−14.7, 7.9]	0.553
Offers implants^a^	91.82	91.81	93.13	89.24	3.89	[−0.4, 8.1]	0.072
Stock‐out of any method offered in the past 3 months^a^	57.61	59.70	50.43	52.84	−0.33	[−18.7, 18.0]	0.972
Family planning outreach services							
Received NGO support^b^	71.33	54.50	56.14	52.82	−13.50	[−33.1, 6.1]	0.177
Received NGO support for CHVs^b^	6.12	2.09	2.58	1.56	−3.00	[−14.7, 8.7]	0.617
Provides family planning through CHVs^e^	42.77	29.75	26.42	18.18	−4.78	[−15.9, 6.4]	0.401
Number of CHVs supported to provide family planning^c^, ^f^	61.95	55.47	67.15	55.42	5.26	[−30.5, 41.0]	0.773
Any mobile outreach visit in the past 12 months^e^	19.25	10.93	20.44	14.17	−2.04	[−16.2, 12.1]	0.777
Number of mobile outreach visits in the past 12 months^d^, ^e^	2.37	3.36	3.86	1.15	**3.69**	**[0.4, 7.0]**	**0.029**
Number of clients served by mobile outreach^c,f^	20.33	4.81	21.42	13.75	−7.85	[−27.5, 11.8]	0.433
LARC offered by mobile outreach^c,f^	12.85	4.57	7.48	4.03	−4.83	[−17.3, 7.7]	0.449
Service integration							
Offers FP and HIV services	87.53	88.04	87.16	81.58	6.09	[−8.5, 20.7]	0.414
Offers FP and PAC services^e^	72.10	72.47	74.87	77.90	−2.65	[−16.5, 11.2]	0.708

NOTE: All models adjusted for facility type, district‐level mCPR, survey year, and standard errors clustered at the enumeration area. Abbreviations: CHV, Community Health Volunteers; FP, family planning; GGR, Global Gag Rule; IUD, intrauterine device; LARC, long‐acting reversible contraception; mCPR, modern contraceptive prevalence rate; NGO, nongovernmental organization; PAC, post‐abortion care; SDPs, service delivery points.

^a^ Excludes pharmacies and retail outlets.

^b^ Stock‐out of any method in the past three months (IUDs, injectables, implants, pills, male condoms, female condoms, emergency contraception).

^c^ Variable only collected in 2018 and 2020, not available for difference‐in‐difference estimates for exposure based on signing organizations.

^d^ Excludes health clinics, pharmacies, and retail outlets.

^e^ Only among SDPs that reported providing FP through CHVs.

^f^ Only among SDPs that reported receiving any mobile outreach.

Our parallel trends analysis revealed no differential trends by exposure status for the contraceptive provision outcomes, support for CHVs, or the service integration outcomes (Supplemental Figures S1–S3). However, divergent trends were observed for the proportion of facilities that received any mobile outreach visits in the past 12 months. Similar proportions of more and less exposed facilities reported receiving visits in the first few rounds of data. Then the gap between these groups of facilities begins to widen after 2016 and continues through 2018, with less exposed facilities experiencing a steeper decline in services. This trend reverses in the post‐GGR period; the decline increases among more exposed facilities and tapers off among facilities less exposed to the GRR such that by 2020 the two exposure groups are once again not statistically different from one another. Given that the trend reversed post‐GGR for this outcome, it may be that our DID model obscures the true impact of noncompliance exposure on any receipt of mobile reach services in our sample.

## DISCUSSION

Overall, our analyses indicate that the GGR likely resulted in negative impacts on family planning service provision in Ethiopia. Our pre–post analysis revealed country‐wide declines in family planning service provision, including reductions in facilities providing family planning through CHVs, mobile outreach, and integrated family planning and PAC services, as well as higher levels of contraceptive stock‐outs. The Pompeo Expansion, and its deep infiltration into the health system, necessitated the use of the pre–post only study design, limiting our ability to make causal inferences. However, previous trends for these outcomes in Ethiopia, the fact that impacts were only observed on services that NGOs play a large role in supporting, and a lack of alternative explanations for the observed changes lead us to conclude that these negative impacts were likely related to the GGR.

Contrary to our hypothesis, we did not observe significant results from most of our DID models estimating the impact of facilities’ exposure to noncompliant organizations. One reason for this is that the bureaucratic process of implementation did not require organizations to immediately comply with the policy, allowing them to continue programs or retain U.S. Government funding through the end of 2018. In addition, early impacts were likely mitigated by donors stepping in to provide stopgap funding (PAI 2018). It is therefore difficult to isolate the GGR's impact on noncompliant organizations, given that the GGR‐related losses in U.S. Government funding were largely offset by stopgap funding.

The one significant result from our noncompliant exposure analysis was an increase in the number of mobile outreach visits among facilities that received any mobile outreach. However, we also found that exposed facilities experienced a (nonstatistically significant) decrease in any mobile outreach visits post‐GGR. It is possible that this decrease in mobile outreach overall led to an increase in the number of mobile outreach visits in areas that were able to maintain this service. As such, we cannot conclude that refusals to sign the GGR led to more mobile outreach visits.

One of our most robust findings from the pre–post analysis is the reduction in CHVs supported to provide family planning services. It is clear from the 2014–2017 national trends that CHV support was steadily increasing prior to our study period, and our observed decrease is in direct opposition to this trend. Further, a similar reduction in CHVs was observed in a companion study conducted in Uganda between 2017 and 2018, which found that the GGR was associated with a significant reduction in CHVs engaged in family planning provision among facilities more exposed to the policy (Giorgio et al. 2020). One possible explanation for this finding is the critical role that NGOs play in supporting public sector family planning service delivery in Ethiopia, including technical assistance and training for public health workers, including CHVs.

There is evidence to suggest that the other significant findings in the pre–post analysis are likely attributable to the GGR. NGOs play an important role in providing and/or supporting PAC in both the private and public sectors, which could explain the observed decline in reported integrated PAC and family planning services post‐GGR. While PAC is not subject to GGR restrictions, its perceived proximity to SAC could make NGOs and/or providers hesitant to provide PAC services. The existence of this type of chilling effect, where organizations overimplemented the restrictions of the GGR out of an abundance of caution, has been highlighted by a recent qualitative report on GGR impacts in Ethiopia (PAI 2018), as well as documented globally at the multinational level (McGovern et al. 2020).

Our observed pre–post reduction in mobile outreach is supported by the U.S. Government's own review of the policy's impacts, which noted that mobile outreach services in Ethiopia were particularly impacted by the GGR, as other organizations were unable to fill this gap in service provision (U. S. Government Accountability Office 2020). We also observed an increase in contraceptive method stock‐outs. While stock‐outs are influenced by a range of factors that impact the commodity supply chain, USAID was providing 73% of public sector contraceptives in Ethiopia prior to the GGR's implementation (PAI 2018). Under the GGR, noncomplaint organizations cannot receive in‐kind support from compliant organizations, including contraceptive commodities. The sheer scope of activities subject to GGR restrictions makes it likely that the policy impacted commodity availability.

Further, we did not identify other large changes in family planning service provision that occurred during this same period. In fact, funding for SRH in Ethiopia increased from other donors post‐GGR (Foreign, Commonwealth & Development Office 2017). While there were changes in the political administration in Ethiopia during this period, we were not able to identify any impact this had on family planning funding, policies, or regulations. This evidence, coupled with the fact that these findings are supported by prior research documenting the negative impacts of the GGR on contraceptive availability (Jones 2015; Ravaoarisoa et al. 2020; Ushie et al. 2020), suggests that the observed declines in family planning services are likely a result of the GGR.

This study has several limitation sassociated with the sample of facilities. Due to the PMA 2020 sampling design, which did not oversample private facilities, the sample for this analysis is predominantly comprised of public facilities. As such, our analysis may be missing changes occurring in family planning service delivery at NGO and private facilities, where the impact of the GGR may be more pronounced. In addition, our sample is not nationally representative. The 2020 sampling strategy only covered six of the major regions of Ethiopia. While 90% of the population lives in the six study regions (Central Statistics Agency 2013), is it possible that there were heterogeneous impacts of the GGR in the regions that were excluded in this analysis.

In addition to sample limitations, we were limited to investigating outcomes that have been routinely gathered in the PMA surveys, which may have led to an underestimation of GGR impacts. Qualitative studies have documented clear impacts of the GGR on partnerships and the larger advocacy environment, which we are not capturing in this analysis (Tamang et al. 2020). Further, the expansion of the policy to all global health funding could result in impacts on other health services outside of family planning (Sherwood 2020). We were also not able to assess the extent to which organizations are correctly implementing the policy. It is unlikely that a compliant organization would admit to any incorrect implementation of the policy given the financial risk; as such, we assume in our analysis that complying with the GGR resulted in the implementation of the terms of the policy. For example, a qualitative report on the implementation of the GGR within Ethiopia highlighted confusion surrounding the policy, leading to both potential over‐ and under the implementation of the GGR PAI 2018. Under or nonimplementation of the policy would result in our estimates being a potential underestimate of the true impacts of the GGR.

Research has shown that prospectively measuring the impact of a policy can be challenging given the potential for policy changes to interfere with initial study designs (Matthay et al. 2020). This was certainly the case in our study. Our initial approach to measuring GGR impact was based on variation in exposure to the policy within Ethiopia. However, the complicated nature of the policy rollout, mitigating factors, and the later Pompeo Expansion may have complicated our ability to isolate the impact of exposure to noncompliant organizations. With an estimated 77% of USAID family planning, HIV, and maternal and child health funding going to 10 NGOs, the number of prime grantees of U.S. Government funding is not large in Ethiopia; however, each of these NGOs has as many as 20 subgrantees (PAI 2018). As such, after the Pompeo Expansion, we effectively lost all ability to capture geographic variation in exposure to organizations compliant with the policy. While this limited our analyses to assessing only pre–post changes, our inclusion of prior national trends in key outcomes provided additional support for the conclusion that observed pre–post changes may be associated with the GGR.

Our DID analysis may also be subject to selection bias. Because this analysis only captures impacts due to noncompliance with the GGR, we no longer have a purely exogenous treatment assignment; it may be that there are important differences in the provision of family planning services that are related to whether those facilities are supported by or engaged with NGOs that refused to sign the GGR. To investigate this concern, we assessed whether there were important differences in key indicators at baseline by noncompliance exposure status, and we did observe statistically significant differences in the underlying mCPR. It may be that NGOs that chose to be noncompliant with the GGR have also been more effective in increasing mCPR in the districts that they serve. We account for this potential source of bias by controlling for district‐level mCPR in our multivariable DID models. However, there may be other confounding factors that are related to noncompliant exposure status and our main study outcomes that we were unable to measure and control for in our study design.

Finally, there may be longer‐term impacts that are unlikely to be observable within our study time frame; administrative burdens placed on organizations may take time to trickle down to facility‐level impacts, and scaling back training and technical assistance programs will slowly erode the health system over time. Although the GGR has since been repealed, it takes time for organizations to recover and reinstate services, meaning that the impact of the policy may be felt for years after its reversal.

This study also has several strengths. Our analysis utilized existing data (2017/2018) and initiated a panel follow‐up survey (2020), which allowed for a matched, longitudinal dataset of facilities for this analysis. Our study is the first to quantitatively investigate the impact of the expanded GGR in Ethiopia and is one of two existing peer‐reviewed research articles quantitatively examining the immediate impact of the current policy (Giorgio et al. 2020). While previous quantitative studies have been forced to rely on crude measures of GGR exposure (such as a dichotomous measure based on a country's official development assistance for family planning and reproductive health from the U.S. Government (Brooks, Bendavid, and Miller 2019; Bendavid, Avila, and Miller 2011), our study carefully created exposure variables using detailed data from a variety of sources to capture the complex nature of the policy and its changes over time.

## CONCLUSION

The expanded Trump Administration GGR was slow to fully roll out and impact NGOs in Ethiopia, but our longitudinal analysis of health facilities shows that it has been associated with a decline in family planning service provision, including community outreach, mobile outreach, stock‐outs, and integration with PAC services. While non‐U.S. donors have contributed substantial funding to offset the loss of U.S. funding, it has been insufficient and unable to mitigate the full impacts of the policy. Our findings also highlight the critical role that NGOs play in supporting the broader health system in Ethiopia. The GGR has now been rescinded by the Biden Administration, but funding and programming gaps may not be easily remedied, and it is not clear how long it will take for service provision to recover. To ensure that family planning service provision does not remain vulnerable to the changing administrations of the U.S. government, sustainably financed robust health systems that ensure sovereign Governments are able to meet the family planning health needs of their populations are needed.

## CONFLICT OF INTEREST

The authors declare no conflicts of interest.

## ETHICS APPROVAL STATEMENT

The Institutional Review Boards of the Guttmacher Institute, Johns Hopkins Bloomberg School of Public Health, and Addis Ababa University provided ethical approval for the study.

## PATIENT CONSENT STATEMENT

Informed consent to publish anonymized data from the research was obtained from all participants recruited to the study.

## Supporting information

Supporting Information

## Data Availability

The data from service delivery points and female interviewees that support the findings of this study are openly available from Performance Monitoring and Accountability at https://www.pma2020.org/request‐access‐to‐datasets. The exposure classification data collected by the authors and used in this study can be accessed by contacting esully@guttmacher.org .
