# Supplementary material for: Impact of the Trump Administration's Expanded Global Gag Rule Policy on Family Planning Service Provision in Ethiopia
Source: Stud Fam Plann. 2022 May 31;53(2):339–59. doi: 10.1111/sifp.12196 (PMC9328269; doi:10.1111/sifp.12196)

| **Supplemental Table 1. Differences pre/post GGR in key outcomes in Ethiopia (pre=2017, post=2020)** |
| --- |
|  |
| \|  \| **Estimated adjusted proportion/mean in pre-period (2017)** \| **Estimated adjusted proportion/mean in post-period (2020)** \| **Pre/Post difference** \| \| \| \| --- \| --- \| --- \| --- \| --- \| --- \| \|  \| 2020 vs 2017 \| 95% CI \| p-value \| \| **Contraceptive availability** \|  \|  \|  \|  \|  \| \| Offers intrauterine devices* \| 69.3 \| 71.2 \| 1.9 \| [-1.4, 5.2] \| 0.253 \| \| Offers implants* \| 91.3 \| 91.2 \| -0.1 \| [-2.4, 2.3] \| 0.959 \| \| Stock-out of any method offered in the past 3 months† \| **53.1** \| **59.2** \| **6.1** \| **[-0.6, 12.8]** \| **0.075** \| \| **Family Planning Outreach Services** \|  \|  \|  \|  \|  \| \| Received NGO support‡ \| 58.2 \| 53.9 \| -4.3 \| [-10.8, 2.2] \| 0.198 \| \| Received NGO support for CHVs‡ \| 3.6 \| 1.9 \| -1.6 \| [-4.2, 0.9] \| 0.205 \| \| Provides family planning through CHVs* \| **30.1** \| **24.4** \| **-5.6** \| **[-10.2, -1.0]** \| **0.016** \| \| Number of CHVs supported to provide FP**§ \| 73.8 \| 78.4 \| 4.5 \| [-9.2, 18.2] \| 0.517 \| \| Any mobile outreach visit past 12 months* \| **20.7** \| **7.7** \| **-13.1** \| **[-17.8, -8.4]** \| **0.000** \| \| Number of mobile outreach visits past 12 months*^^ \| 2.7 \| 2.2 \| -0.5 \| [-1.8, 0.8] \| 0.462 \| \| Number of clients served by mobile outreach^^‡ \| 25.2 \| 32.5 \| 7.2 \| [-3.3, 17.8] \| 0.179 \| \| LARC offered by mobile outreach^^‡ \| 6.9 \| 5.6 \| -1.3 \| [-4.9, 2.3] \| 0.475 \| \| **Service integration** \|  \|  \|  \|  \|  \| \| Offers FP and HIV services \| 86.5 \| 86.3 \| -0.2 \| [-4.8, 4.3] \| 0.927 \| \| Offers FP and PAC services* \| **77.3** \| **72.5** \| **-4.8** \| **[-9.1, -0.5]** \| **0.028** \|   Notes: All models adjusted for facility type, district-level mCPR, and standard errors clustered at the enumeration area  † Stock-out of any method in the past 3 months (IUDs, injectables, implants, pills, male condoms, female condoms, emergency contraception).  ‡ Variable only collected in 2018 and 2020, and therefore, pre-period defined as 2018, rather than 2017.  § Only among SDPs that reported providing FP through CHVs  ^^ Only among SDPs that reported receiving any mobile outreach  †† The pre-period based on exposure to signing organizations is defined as 2017, and the post-period is 2018-2020  . * excludes pharmacies and retail outlets  ** excludes health clinics, pharmacies, and retail outlets |

**Supplemental Figure 1. Parallel trends in contraceptive provision outcomes between facilities more and less exposed to organizations not complying with the GGR in Ethiopia, 2014-2020**

**
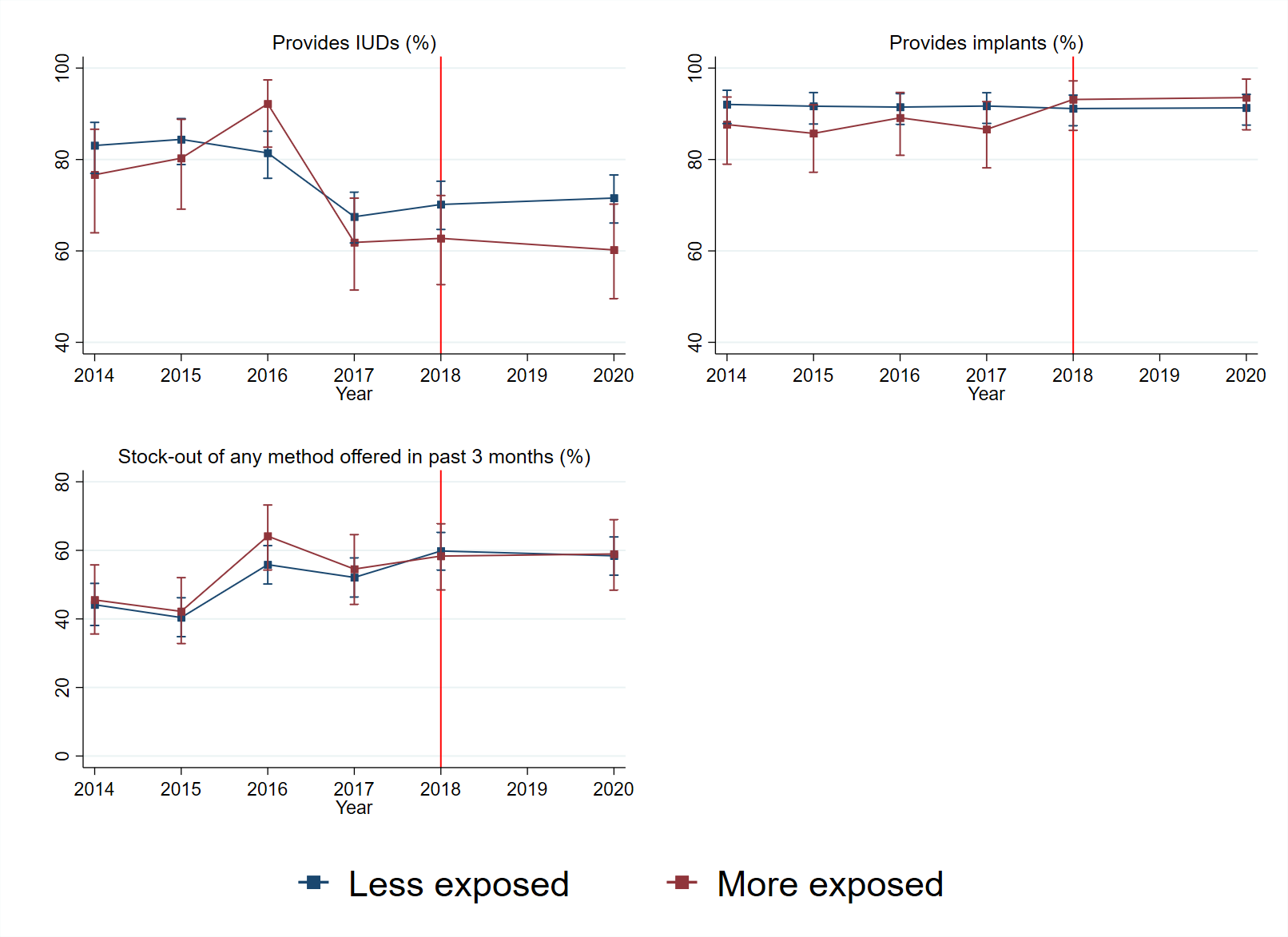
**

**Supplemental Figure 2. Parallel trends in family planning outreach services outcomes between facilities more and less exposed to organizations not complying with the GGR in Ethiopia, 2014-2020**


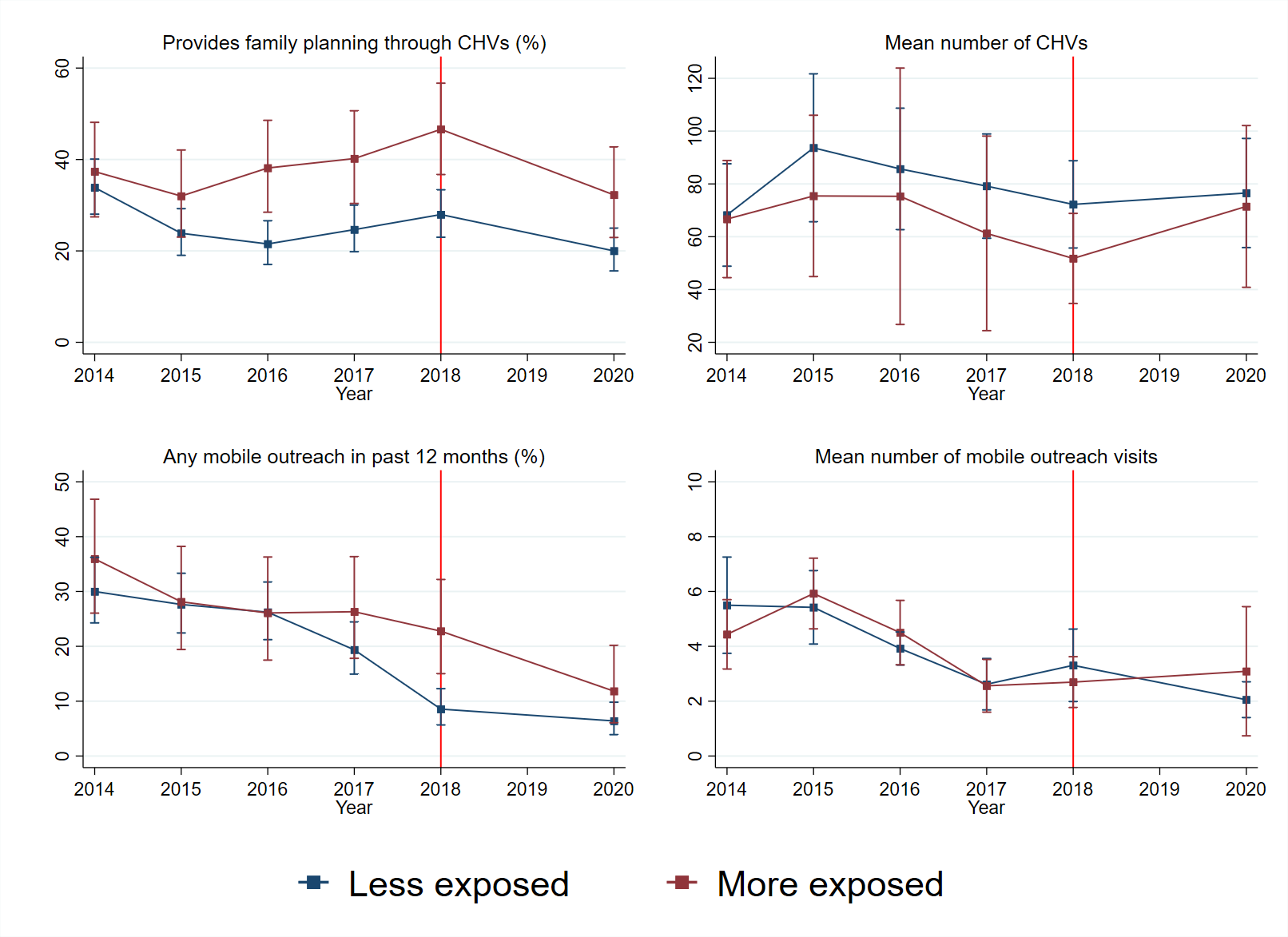


**Supplemental Figure 3. Parallel trends in family planning service integration outcomes between facilities more and less exposed to organizations not complying with the GGR in Ethiopia, 2014-2020**


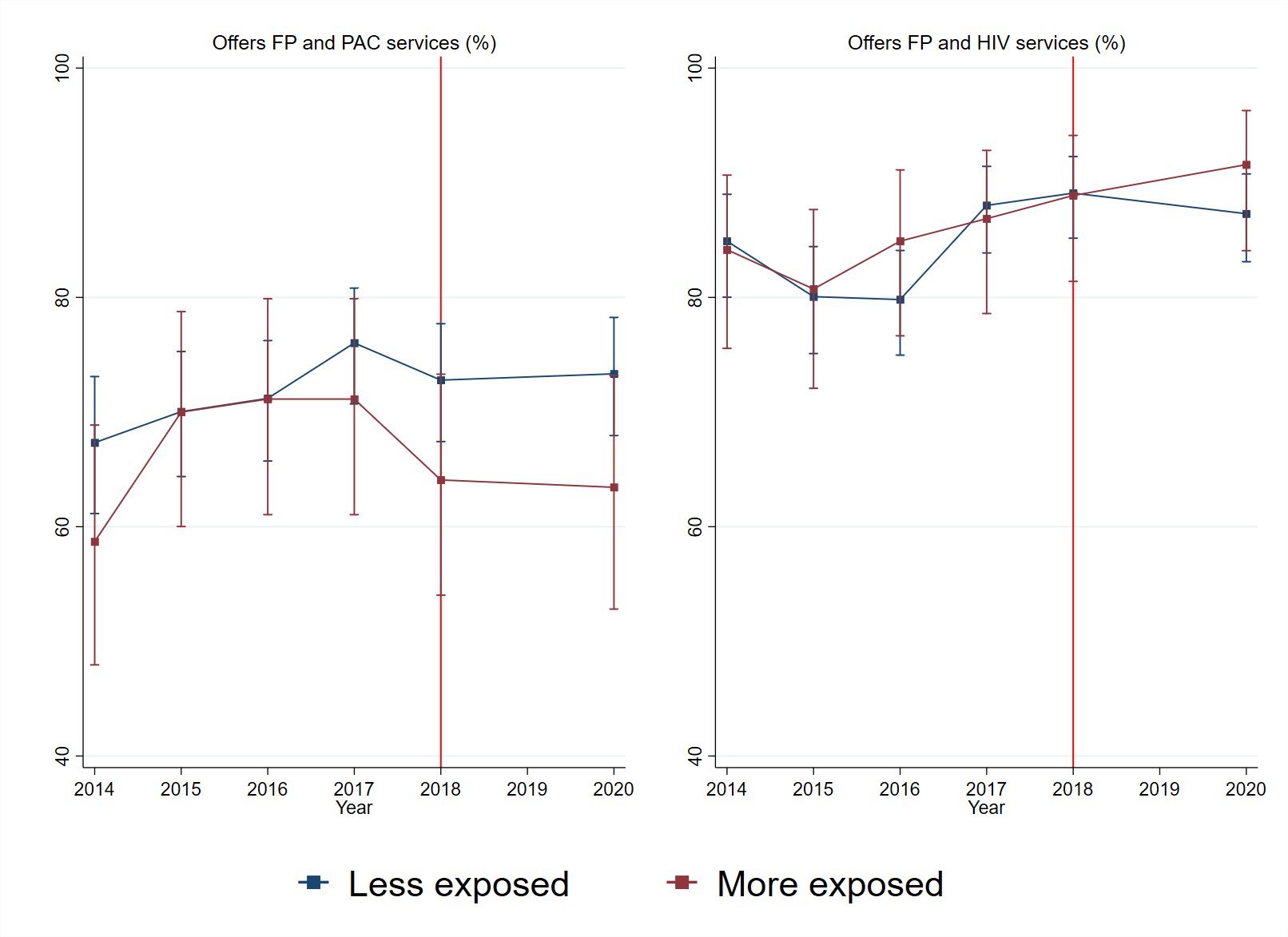

Supplement: Supplementary file 1 — Supporting Information [file SIFP-53-339-s001.docx]
